# Abnormalities of cortical and subcortical spontaneous brain activity unveil mechanisms of disorders of consciousness and prognosis in patients with severe traumatic brain injury

**DOI:** 10.1016/j.ijchp.2024.100528

**Published:** 2024-11-28

**Authors:** Chang Li, Peng Chen, Yongbing Deng, Lei Xia, Xiaodong Wang, Min Wei, Xingdong Wang, Lun Dong, Jun Zhang

**Affiliations:** a Medical Imaging Department, Chongqing Emergency Medical Center, Chongqing University Central Hospital, Chongqing 400014, China; b Department of Neurosurgery, Chongqing Key Laboratory of Emergency Medicine, Chongqing Emergency Medical Center, Chongqing University Central Hospital, Chongqing 400014, China; c Department of Neurosurgery, Xinhua Hospital Affiliated to Shanghai Jiao Tong University School of Medicine, Shanghai 200092, China; d Department of Neurosurgery, Clinical Medical College of Yangzhou University, Yangzhou 225000, China

**Keywords:** Disorders of consciousness, rs-fMRI, Severe traumatic brain injury, Spontaneous brain activity, Prognosis

## Abstract

**Objective:**

To investigate the spatial distribution characteristics of alterations in spontaneous brain activity in severe traumatic brain injury (sTBI) patients with disorders of consciousness (DOC), based on the mesocircuit theoretical framework, and to establish models for predicting recovery of consciousness.

**Methods:**

Resting-state functional magnetic resonance imaging was employed to measure the mean fractional amplitude of low-frequency fluctuations (mfALFF) in sTBI patients with DOC and healthy controls, identifying differential brain regions for conducting gene and functional decoding analyses. Patients were classified into wake and DOC groups according to Extended Glasgow Outcome Score at 6 months. Furthermore, predictive models for consciousness recovery were developed using Nomogram and Linear Support Vector Machine (LSVM) based on mfALFF.

**Results:**

In total, 28 sTBI patients with DOC and 30 healthy controls were included, with no significant baseline differences between groups (*P* > 0.05). The results revealed increased mfALFF of subcortical Ascending Reticular Activating System and decreased cortical mfALFF (default mode network) in DOC patients within the framework of the mesocircuit model (FDR_*P* < 0.001, Clusters > 100). The study identified 2080 differentially expressed genes associated with reduced brain activity regions, indicating mechanisms involving synaptic function, the oxytocin signaling pathway, and GABAergic processes in DOC formation. In addition, significantly higher mfALFF values were observed in the left angular gyrus, supramarginal gyrus, and inferior parietal lobule of DOC group compared to the wake group (AlphaSim_*P* < 0.01, Cluster > 19). The Nomogram prediction model highlighted the pivotal role of these regions' activity levels in prognosis (AUC = 0.90). Validation using LSVM demonstrated robust predictive performance with an AUC of 0.90 and positive predictive values of 80% for wake and 83% for DOC.

**Conclusions:**

This study offered crucial insights underlying DOC in sTBI patients, demonstrating the dissociation between cortical and subcortical brain activities. The findings supported the use of mfALFF as a robust and non-invasive biomarker for evaluating brain function and predicting recovery outcomes.

## Introduction

Severe traumatic brain injury (sTBI), high morbidity, mortality, and disability, is a significant global public health concern (Maas et al., 2022). Managing patients with sTBI often presents the challenge of navigating a complex condition and making decisions amidst variability (Maas et al., 2017; Rosenfeld et al., 2012). After overcoming the initial challenges, most survivors who suffer from sTBI are likely to become long-term disorders of consciousness (DOC), severe disability, so it is necessary to find appropriate prognostic predictors (Lingsma et al., 2010). Specifically, DOC after sTBI includes acute coma, as well as long-term impaired consciousness, which can be permanent (Katz et al., 2009). Although early rehabilitation is most helpful, uncertain rehabilitation potential affects care and treatment decisions. However, it is the uncertainty of this outcome that fuels the interest in exploration. At present, it has been found that biomarkers have important clinical study value in predicting the prognosis of sTBI (Gosseries et al., 2016). However, access to most markers is either invasive, indirectly characterised indicators or unstable (Ji et al., 2015; Zhang et al., 2021). Furthermore, most markers remain at the level of predicting survival or non-survival, with insufficient predictive power regarding awakening.

The fundamental aspect of traumatic DOC is an aberrant conduction of impulses within the neuronal pathways, coupled with a disruption in the conscious circuit projections. Therefore, detection of residual brain function and circuits may be a potential measure to reveal the mechanisms and prognosis of DOC. The emerging resting-state functional magnetic resonance imaging (rs-fMRI) technology has brought new methods for non-invasive monitoring of brain function. With the development of methodology, the mean fractional amplitude of low frequency fluctuations (mfALFF) is proposed, laying the foundation for the characterization of spontaneous brain activity. The use of mfALFF to represent spontaneous brain activities in neurological diseases has good reproducibility and application prospects (Jia et al., 2020; Yu et al., 2022; Zeng et al., 2023; H. Zhang et al., 2019).

The evidence from resting-state functional connectivity (FC) indicates that sTBI patients with DOC is associated with alterations in brain network-scale mechanisms. Disruption of mesocircuit signalling with the thalamus as the hub is a potential mechanism for DOC (Thibaut et al., 2019; J. Zhang, H. Zhang, et al., 2022). The mesocircuit model theory proposes a framework for explaining the interactions between the cortical and subcortical structures in regulating consciousness. According to this theory, the interaction of information from cortical and subcortical structures forms the mesocircuit model of consciousness (Annen et al., 2023; Schiff, 2010; Thibaut et al., 2019; J. Zhang, H. Zhang, et al., 2022). This circuit includes the frontal lobe, parietal lobe, brainstem, and thalamus, which are interconnected through complex neural networks to coordinate arousal and awareness (Annen et al., 2023; Schiff, 2010; Thibaut et al., 2019; J. Zhang, H. Zhang, et al., 2022). The mesocircuit model theory provides a comprehensive perspective for understanding the neural mechanisms underlying the formation and regulation of consciousness. Subsequent mechanistic studies and neuromodulation arousal practice validated the mesocircuit model (Chudy et al., 2023; Yang et al., 2023). Previous researches indicated that the failure to recover consciousness was correlated with the losses of subcortical fronto-temporoparietal connectivity and excitatory corticothalamic synaptic strength (Panda et al., 2022; Tewarie et al., 2024). The loss of consciousness was associated with the diminished network interactions, the alterations in the hemodynamic response within the default mode network (DMN), as well as the impairment of posterior integration and thalamo-frontotemporal broadcasting (Lopez-Gonzalez et al., 2021; Panda et al., 2023; Wu et al., 2019). Injury remodelling is the result of brain injury with brain dysfunction and compensation of residual brain function. However, the characteristics and spatial distribution patterns of brain activity and topological properties in each module or unit within the mesocircuit remain unclear after sTBI. This will significantly advance our understanding of DOC mechanisms, contributing to unraveling prognosis and providing insights for neuromodulation interventions.

Thus, this study applied mfALFF to assess the level of spontaneous brain activity in patients based on the mesocircuit theoretical framework, with the main aim of investigating the brain activity characteristics of each structural unit and contributing to further elucidation of the mechanism of DOC formation after sTBI. The research includes finding the differential brain regions of mfALFF of patients with sTBI compared with healthy controls, as well as related gene and functional decoding. In addition, the models for predicting recovery of consciousness in sTBI based on the mfALFF were established.

## Methods

### Participants

The current study was designed in accordance with the Strengthening the Reporting of Observational Studies in Epidemiology (STROBE) guidelines (Vandenbroucke et al., 2007) and received approval from the Hospital Ethics Committee (2020KY-179). All subjects were recruited at our neurology center from June 2020 to June 2021. A total of 30 healthy controls (20 males and 10 females), were included in this study. Finally, a total of 28 sTBI patients with DOC were included, consisting of 19 males and 9 females.

Inclusion criteria: (1) Age 18 to 80 years; (2) sTBI (Glasgow Coma Scale, GCS ≤ 8 points), the diagnosis and degree were established by the 4th, Brain Trauma Foundation Guidelines (Carney et al., 2017). The GCS was used to assess a patient's level of consciousness based on three criteria: eye opening (1-4 score), verbal response (1-5 score), and motor response (1-6 score); (3) In the 1st to 4th weeks after sTBI; (3) No history of neuropsychiatric disorders prior to the onset; (4) Normal work communication before sTBI; 5) Right-handedness; 6) Stable vital signs and spontaneous breathing; and 7) The family was informed and signed a consent form.

Exclusion criteria: 1) Circulatory failure (unresponsive to vasopressor agents) or respiratory failure (require assisted ventilation); 2) Severe systemic polytrauma; 3) Combined with hemolysis, hemorrhagic shock, seizure, and renal failure; 4) Pregnant or lactating; 5) Contraindications to MRI; and 6) History of drug and alcohol abuse.

### rs-fMRI parameters

rs-fMRI scans was performed by using a 3.0 Tesla system (Discovery MR750; GE Medical Systems, Milwaukee, WI, USA) with gradient echo planar (TR = 2000 ms, TE = 30 ms, flip angle = 90^o^, slice thickness = 3.5 mm). Meanwhile, a 3D T1-weighted image was acquired covering the whole brain (TR = 8.16 ms, TE = 3.18 ms, flip angle =12°, slice thickness = 1 mm).

During the rs-fMRI scan, both sTBI patients and healthy participants were positioned supine with their heads securely immobilized by sponges and a coil to reduce head movement. MRI-compatible monitors were employed to oversee the vital signs of all participants, with scanning to be discontinued immediately in the event of any abnormalities.

### Outcome assessment

All patients were managed according to the current institutional protocols by a dedicated team of neurosurgeons holding senior professional. Prior to scans, all scheduled patients refrained from using benzodiazepines and central α2 receptor agonists for 8 hours (Dexmedetomidine was widely in our Neurosurgery Center). Management of elevated intracranial pressure was conducted without intracranial pressure monitoring devices, utilizing mannitol (every 6-8 hours) and/or hypertonic saline (3%, 250-500 ml). Complications such as hypoxia, hypotension, acidosis, and pneumonia were addressed following a precise protocol for all patients. Those with stabilized conditions were subsequently transferred to a designated rehabilitation center for systematic awakening and rehabilitation therapy.

A professionally trained neurosurgeon, blinded to the study's details, assessed the neurological prognosis outcomes of DOC patients at 6-month through telephone interviews with close family members and/or outpatient follow-up visits. The prognosis of DOC patients was evaluated using the Extended Glasgow Outcome Score (GOS-E) (Okonkwo et al., 2013). GOS-E was an 8-point ordinal scale: 1 = Death, 2 = Vegetative State, 3 = Lower Severe Disability, 4 = Upper Severe Disability, 5 = Lower Moderate Disability, 6 = Upper Moderate Disability, 7 = Lower Good Recovery, and 8 = Upper Good Recovery. Patients with a GOS-E score of 3 or higher were classified as the wake group, while those with a GOS-E score of 2 were categorized as the DOC group (Zhang et al., 2022).

### fMRI data analysis

Pre-processing of the rs-fMRI data was performed by using DPABI V7.0 toolbox (http://rfmri.org/DPABI) written in Matlab 2016b (https://www.mathworks.com/). The pipeline included (Zhang et al., 2023): (1) Conversion of the DICOM data to NIFTI format; (2) Removal of the first 10 volumes (i.e. first 10 time points); (3) Slice timing correction; (4) Realignment to correct for head motion (participants with any translation > 2.0 mm and/or rotation > 2° were excluded); (5) Segment the structure image into gray matter, white matter, and cerebrospinal fluid; (6) Normalize image to Montreal standard head anatomic space (reslice by 3 × 3 × 3 mm); (7) Linear detrending of potential drift in image signal intensity; 8) Bandpass filtering (0.01-0.08 Hz). In addition, the so-called Friston 24 parameter head motion model was applied to regress out remaining noise due to head motion (Satterthwaite et al., 2013) and nuisance (e.g., cerebrospinal fluid and white matter). Finally, spatial smoothing was performed with a Gaussian kernel (FWHM = 4 × 4 × 4 mm^3^).

The time series of each voxel was transformed to the frequency domain and then the power spectrum was then obtained. After calculated the square root at each frequency of the power spectrum, the averaged square root of power spectrum within 0.01-0.08 Hz frequency band was retained as the ALFF (Zang et al., 2007). The fALFF was defined as a ratio of the power of each frequency at the low-frequency range (0.01-0.08 Hz) to that of the entire detectable frequency range (0-0.25 Hz) (Zou et al., 2008). For standardization, the mfALFF maps were obtained from that fALFF divided by global voxel-wise in individual entire brain volume (Ho et al., 2021).

A two-sample t-test with covariate adjustment (ANCOVA) was used to compare the differences in whole-brain mfALFF between sTBI patients with DOC and healthy controls, as well as between the wake and DOC groups (adjusted covariates cerebrospinal fluid, white matter, head motion coefficient, age, and gender). We conducted multiple comparison correction using False Discovery Rate (FDR) (q < 0.001) or AlphaSim (*P* < 0.05, clusters > 154) to adjust the results. Using the peak coordinates of the differential brain regions between sTBI patients and healthy controls, we extracted corresponding clusters as regions of interest (ROIs). These ROIs were then analyzed for their topological properties. In addition, the study applied the wake and DOC group difference brain regions as ROI and extracting mfALFF values for quantitative and prognostic assessment analyses.

The study constructed a ROI-ROI brain network FC matrix (r values) and utilized the Gretna software (https://www.nitrc.org/projects/gretna/) to calculate the topological properties of each ROI, including degree centrality (DC) and node efficiency (Ne). Specifically, we set a lower bound of 0.2 “[r, max]=gretna_get_rmax (load ('xxx.txt'))”, with a step size of 0.01 and a maximum value of 0.5, to establish a framework for analyzing the topological properties with a sparsity level of 31. The area under the curve (AUC) corresponding to the metrics (aDC and aNe) at all sparsity levels was used for intergroup comparisons, applying FDR correction (q < 0.05) for statistical significance.

### Gene expression analysis of abnormal brain regions

The Allen Human Brain Atlas (AHBA), is an epic gene expression atlas compiled at the Allen Institute for Brain Sciences (https://human.brain-map.org/) and which is normalized to the MNI template. The criteria that were applied in recruiting donors to allow construction of the atlas were as follows (Hawrylycz et al., 2012): 1) age 18-68 years; 2) no history of neurological disorder, psychiatric disorder, substance abuse or addiction; 3) consent and authorization from next-of-kin; and 4) no chronic medical history or diagnosis of severe ischemic-hypoxic encephalopathy. The AHBA microarray gene expression atlas, was constructed from tissue samples taken bilaterally in two, and from the left cerebral hemisphere of four, post-mortem brains obtained from six donors who died of non-neurological diseases (Hawrylycz et al., 2012). The six (5 male, 1 female) adult donors included 3 Caucasian, 2 African and 1 Hispanic, aged 24-57 years (Hawrylycz et al., 2012).

Following quality control the normalized microarray data were pre-processed by using the AHBA data cleaning pipeline (http://help.brain-map.org/display/humanbrain/documentation/), which includes probe to gene annotation corroboration, probe cleaning and selection steps (Hawrylycz et al., 2012; Z. Liu et al., 2019; Marcus et al., 2013; Sunkin et al., 2013) according to the optimized scheme reported by Liu et al. (2019). Due to the multiple correspondence of probes with individual genes, by using the 'collapserws' function in the WGCNA package the probe with the highest average expression value as used to characterize the corresponding gene. Accordingly, a total of 20,738 microarray gene expression profiles and 3,695 matched brain tissue samples were identified. Next, the genetic data were normalized in a manner which superimposed group-level effects and reduced individual variation. Subsequently, a spherical ROI (6 mm) was constructed for each of the 3,695 tissue samples using their MNI center-of-mass coordinates as the origin. Finally, the Human Connectome Workbench software was used to map these data onto the Conte 69 atlas.

More than half of the voxels in the whole brain tissue mask are represented in the AHBA samples. The abnormal brain regions (DOC patients *vs*. healthy controls) were compared to regions of expression in the gene atlas, and a permutation test was used to detect significant differences in gene expression between the abnormal brain regions and randomly sampled specimens (10,000 replicates, *P* < 0.001).

### Gene enrichment analysis

Enrichment analysis of overexpressed genes was performed by using the online workbench known as Metascape (https://metascape.org/gp/index.html) (Zhou et al., 2019). The following procedure was applied: (1) transform gene identifiers into Entrez gene IDs; (2) add annotations to genes (e.g. gene function, gene type, gene description and protein classification); (3) perform cluster analysis of listed genes; (4) perform enrichment analysis of the full Metascape database for a given genes; and (5) produce a report containing enrichment graph and table of results. The focus of the present study was on biological functions of genes, pathways of action, cell type characteristics, and disease enrichment.

### Function decoding of abnormal brain regions

The study utilized Neurosynth (https://www.neurosynth.org/) to perform brain region functional decoding analysis. Neurosynth features voxel-scale functional terms and task decoding capabilities (containing a large number of task activation maps), which can automatically integrate and analyze functional imaging data based on big data (approximately 11,000 research studies). This section aims to identify the functional attributes of abnormal spontaneous brain activity regions in DOC patients compared to healthy controls and corroborate these findings with gene enrichment results.

### Statistical analysis

All statistical analysis was performed by using SPSS 26 (https://www.ibm.com/products/spss-statistics) and GraphPad Prism 9.5 (https://www.graphpad.com/). Initially, Kolmogorov-Smirnov tests were applied to examine the normality of the distribution for each continuous variable. If the distribution was normal, inter-group comparison was performed by using a two-sample t-test (mean ± standard deviation). Otherwise, a Mann-Whitney U test was used (median and quartiles). For categorical variables a Chi-square test was used.

Univariate binary logistic regression analysis based on the mfALFF values corresponding to ROI in different prognostic groups was performed using R 4.0 (https://www.r-project.org/). The Nomogram prediction model was implemented using the 'rms' package in R 4.0 (Chen et al., 2022). Next, the 'rms', and 'pROC', packages were respectively used to perform receiver operating characteristic curve (ROC) analyses for the purposes of validation and discrimination testing. An AUC of 0.5 indicates random outcome, while an AUC of 1 represents perfect discriminative capability.

To validate the robustness of ROI in predicting the prognosis of DOC patients, the Linear Support Vector Machine (LSVM) (2-fold crossover) analysis was implemented in the machine learning platform in Matlab 2016b. LSVM is a well established supervised learning method based on classification (Stafford et al., 2020). The main objective is to construct a boundary in the dataset to separate different categories of data (Stafford et al., 2020). LSVM has been shown to have better performance and higher computational efficiency compared with traditional classification methods (Stafford et al., 2020).

## Results

### Baseline characteristics

A total of 58 participants were enrolled in the study. The 30 subjects (20 male) were in the healthy control group with an average age of 56.6 years and were all right-handed. The 28 DOC patients (19 male) with an mean age of 58.0 years were all right-handed. There were no differences in age, gender, and right-handedness between the two groups (*P* > 0.05). In addition, seven of the 28 DOC patients died after 6 months. The remaining 21 patients were divided into two groups: 13 patients were in wake group and 8 patients were also in DOC group. The average admission GCS of 28 patients with DOC was 5 and the GOS-E was 3 after 6-month. The mean admission GCS of DOC group was 2.88, which was statistically lower than that in wake group (admission GCS was 5.85) (*P* = 0.002). Similarly, The average GOS-E of patients in DOC group was 2.00 which was statistically lower than that in wake group (GOS-E = 4.69) (*P* < 0.001). The baseline characteristics of patients were detailed in Table 1 and **Supplementary table**.

**Table 1:** Baseline characteristics of the included subjects.

**Characteristic**	**Healthy controls**	**DOC patients**	**Wake group**	**DOC group**	***P-*value**
**Age, (Mean ± SD)**	56.60 ± 18.24	58.00 ± 15.39	52.23 ± 17.22	59.38 ± 15.98	0.75^a^; 0.36^b^
**Gender, male (%)**	20 (66.7)	19 (67.9)	8 (61.5)	7 (87.5)	0.92^a^; 0.20^b^
**Right-handed, N (%)**	30 (100.0)	28 (100.0)	13 (100.0)	8 (100.0)	-
**aGCS, (Mean ± SD)**	-	5.00 ± 2.45	5.85 ± 2.38	2.88 ± 1.36	0.002^b^
**GOS-E, (Mean ± SD)**	-	3.00 ± 1.93	4.69 ± 1.49	2.00 ± 0.00	< 0.001^b^

DOC, disorders of consciousness; aGCS, admission Glasgow Coma Score;

GOS-E, Extended Glasgow Outcome Score; (a. controls *vs.* DOC; b. wake group *vs.* DOC group).

### Spontaneous brain activity reveals DOC mechanisms

**Fig. 1:**
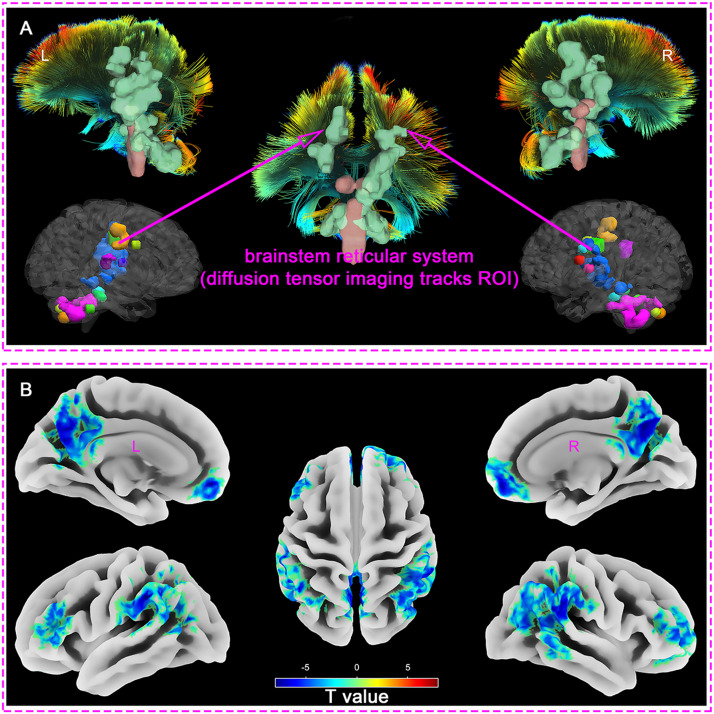
Regions of abnormal spontaneous brain activity in DOC patients relative to healthy controls (L, left; R, right; A. Regions of elevated spontaneous brain activity; B. Regions of reduced spontaneous brain activity; ROI, region of interest).

Measuring the mfALFF values of different brain regions in DOC patients compared with healthy controls, we found that the regions with enhanced mfALFF included: Cerebellar Tonsil, Cerebellum Posterior Lobe, Cerebellum Anterior Lobe, Brainstem, Thalamus, Globus Pallidus, Limbic Lobe, Insula, and Paracentral Lobule (FDR_q < 0.001, Clusters > 100, Table 2 and **Supplementary material 1**). The findings indicated that regions of enhanced spontaneous brain activities in the brainstem, thalamus, limbic system, and some cortical regions were highly overlapped with the Ascending Reticular Activating System (ARAS) (Fig. 1**. A**).

**Table 2:** Brain regions with abnormal spontaneous brain activity in DOC patients relative to healthy controls.

**Volexs**	**Regions**	**Peak MNI x y z**	**T value**	**q-value**
enhanced				
382	Cerebellar Tonsil, Cerebellum Posterior Lobe, Cerebellum Anterior Lobe, Brainstem, Uvula,	6, -39, -33	9.24	FDR < 0.001
226	Extra-Nuclear, Midbrain, Thalamus, Cerebellum Anterior Lobe, Lentiform Nucleus, Insula, Putamen, Ventral Posterior Lateral Nucleus, Globus Pallidus, Precentral Gyrus, Postcentral Gyrus, Cingulate Gyrus, Inferior Parietal Lobule, Frontal Lobe	-6, -21, -12	7.48	FDR < 0.001
128	Extra-Nuclear, Limbic Lobe, Cingulate Gyrus, Insula, Medial Frontal Gyrus, Supplementary Motor Area, Inferior Parietal Lobule, Paracentral Lobule, Thalamus	30, -27, 21	5.78	FDR < 0.001
weakened				
407	Superior Frontal Gyrus, Medial Frontal Gyrus, Inferior Frontal Gyrus, , Brodmann area 10, Orbital Gyrus, Brodmann area 46	3, 57, -15	-8.17	FDR < 0.001
906	Inferior Parietal Lobule, SupraMarginal, Brodmann area 40, Angular, Superior Temporal Gyrus, Middle Temporal Gyrus, Brodmann area 39, Postcentral Gyrus, Superior Parietal Lobule, Precuneus, Inferior Temporal Gyrus	60, -42, 36	-7.71	FDR < 0.001
155	Middle Frontal Gyrus, Inferior Frontal Gyrus, Brodmann area 46, Brodmann area 10	-45, 39, 21	-5.82	FDR < 0.001
858	Precuneus, Limbic Lobe, Brodmann area 7, Posterior Cingulate, Brodmann area 31, Cingulate Gyrus,	0, -60, 33	-8.24	FDR < 0.001
313	Inferior Parietal Lobule, Brodmann area 40, Supramarginal Gyrus, Superior Temporal Gyrus, Postcentral Gyrus, Brodmann area 39, Middle Temporal Gyrus	-63, -36, 33	-9.45	FDR < 0.001
101	Angular, Middle occipital gyrus, Inferior Parietal Lobule, Brodmann area 39, Superior Parietal Lobule, Middle Temporal Gyrus, Precuneus	-39, -75, 24	-6.58	FDR < 0.001

MNI, Montreal Neurological Institute; FDR, False Discovery Rate

Compared with healthy controls group, the mfALFF of DOC patients weakened in the following regions: Superior Frontal Gyrus, Medial Frontal Gyrus, Inferior Frontal Gyrus, Brodmann area 10, Brodmann area 46, Inferior Parietal Lobule, Superior Parietal Lobule, Supramarginal Gyrus, Angular, Superior Temporal Gyrus, Middle Temporal Gyrus, Inferior Temporal Gyrus, Precuneus, and Posterior Cingulate (FDR_q < 0.001, Clusters > 100, Table 2 and **Supplementary material 2**). The results revealed that the regions with reduced spontaneous brain activities were highly consistent with the default mode network (DMN) (Fig. 1**. B**).

### Making ROIs based on abnormal areas of spontaneous brain activity in DOC patients

**Fig. 2:**
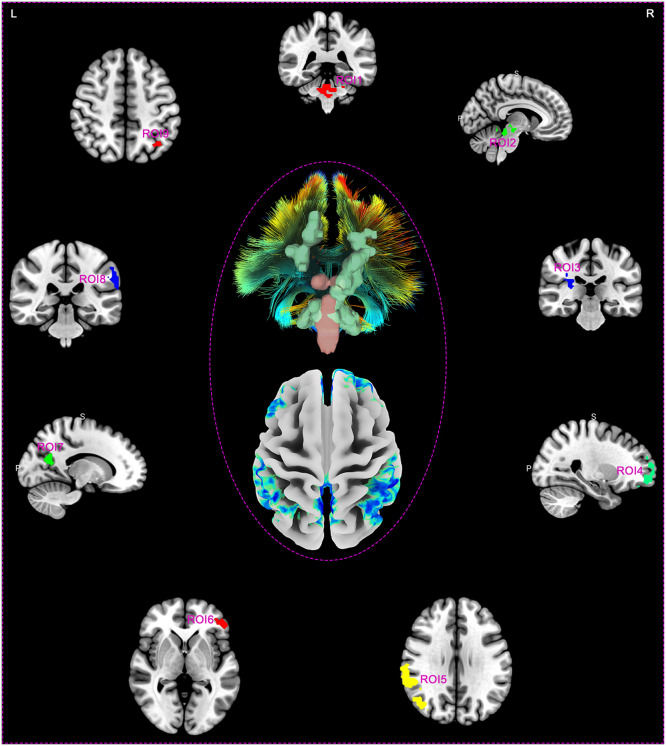
Masks created based on the peak coordinate points of abnormal spontaneous brain activity in DOC patients (ROI, region of interest).

DOC patients exhibited nine clusters of spontaneous brain activity differences centered around peak coordinate points, compared to healthy controls. Fig. 2 illustrates the brain region distribution map of these 9 ROIs, which includes 3 regions with enhanced spontaneous brain activity (ROI1, ROI2, and ROI3), while the remaining 6 areas (ROI4, ROI5, ROI6, ROI7, ROI8, and ROI9) displayed reduced spontaneous brain activity. Specifically, the ROIs correspond to brain regions ROI1 (x/y/z 6/-39/-33; Cerebellum Posterior Lobe, Cerebellum Anterior Lobe, Brainstem, and Pons), ROI2 (x/y/z -6/-21/-12; Midbrain, Thalamus, and Cerebellum Anterior Lobe), ROI3 (x/y/z 30/-27/21; Limbic Lobe, Cingulate Gyrus, and Insula), ROI4 (x/y/z 3/57/-15; Medial Frontal Gyrus, and Superior Frontal Gyrus), ROI5 (x/y/z 60/-42/36; Inferior Parietal Lobule, Brodmann area 40, Angular, Middle Temporal Gyrus, Postcentral Gyrus, and Precuneus), ROI6 (x/y/z -45/39/21; Middle Frontal Gyrus, Inferior Frontal Gyrus, and Brodmann area 46), ROI7 (x/y/z 0/-60/33; Precuneus, Limbic Lobe, and Posterior Cingulate), ROI8 (x/y/z -63/-36/33; Inferior Parietal Lobule, Supramarginal Gyrus, Superior Temporal Gyrus, Postcentral Gyrus, and Angular), and ROI9 (x/y/z -39/-75/24; Angular, Inferior Parietal Lobule, Superior Parietal Lobule, Middle Temporal Gyrus, and Precuneus), respectively (**Supplementary material 1 and 2**).

### DC properties of DOC patients

**Fig. 3:**
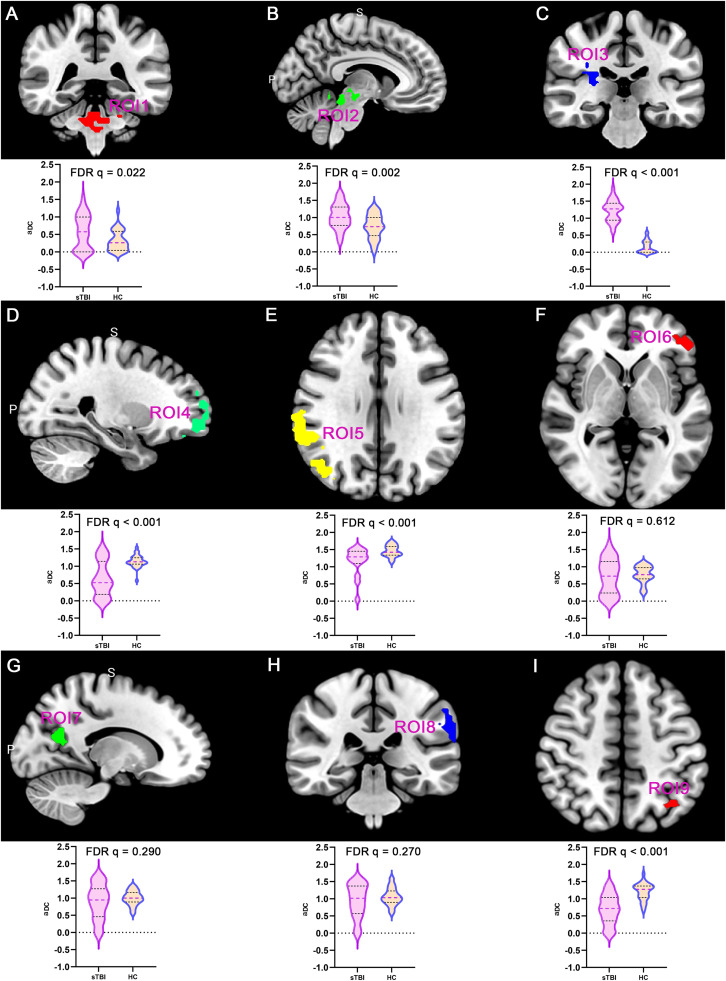
Alterations in aDC value of ROIs in DOC patients compared to healthy controls (ROI, region of interest; FDR, False Discovery Rate; aDC, area under the curve-degree centrality; sTBI, severe traumatic brain injury; HC, health control).

This study analyzed the DC topological properties of the aforementioned 9 ROIs in DOC patients, with the results presented in Fig. 3. In the patients with DOC, the aDC values of ROI1, ROI2 and ROI3 were enhanced in comparison to the healthy controls (FDR q < 0.05). However, the aDC values for ROI 4, ROI 5, and ROI 9 were significantly lower in the DOC patients (FDR q < 0.05). The findings indicate that following sTBI, the number of FC in ARAS-derived ROIs significantly enhanced (as hub nodes), while DMN-derived ROIs were reconfigured as non-hub nodes. This finding closely aligns with the spatial distribution patterns of spontaneous brain activity previously described.

### Ne properties of DOC patients

**Fig. 4:**
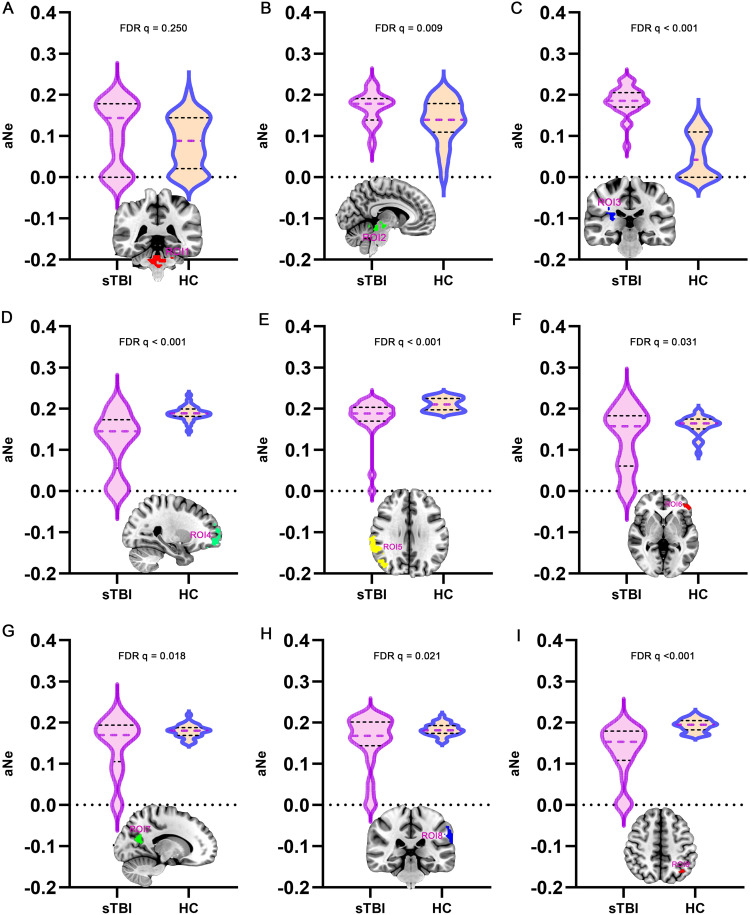
Alterations in aNe value of ROIs in DOC patients relative to healthy controls (ROI, region of interest; FDR, False Discovery Rate; aNe, area under the curve-node efficiency; sTBI, severe traumatic brain injury; HC, health control).

Furthermore, in the brain network formed by 9 ROIs, the Ne was analyzed as one of the topological properties (Fig. 4). The aNe corresponding to ROI2 and ROI3 in DOC patients was significantly enhanced, compared to healthy controls (FDR q < 0.05). In DOC patients, the aNe of brain regions ROI4, ROI5, ROI6, ROI7, ROI8, and ROI9 was significantly reduced, with statistically significant differences (FDR q < 0.05). The above results suggest that after sTBI, the Ne of subcortical ARAS-derived ROIs increases compensatorily in an attempt to enhance arousal, while the Ne values of cortical DMN-derived ROIs decreases, which may be closely related to deficits in awareness.

### Gene and functional decoding in brain regions with weakened spontaneous brain activities

According to the mfALFF enhanced brain regions, it was recognized that the ARAS played a critical role in DOC. Furthermore, in view of the fact that the regions of weakened spontaneous brain activities were highly consistent with the spatial distribution of the typical DMN, it was revealed that these regions might closely related to the mechanisms of DOC. This study further combined the regions with weakened spontaneous brain activities with Allen Human Brain Atlas to find differentially expressed genes, and tried to clarify the underlying biomolecular mechanism of DOC through gene enrichment analysis. Gene enrichment analysis was mainly performed through the Metascape database, and the results were as follows.

**Fig. 5:**
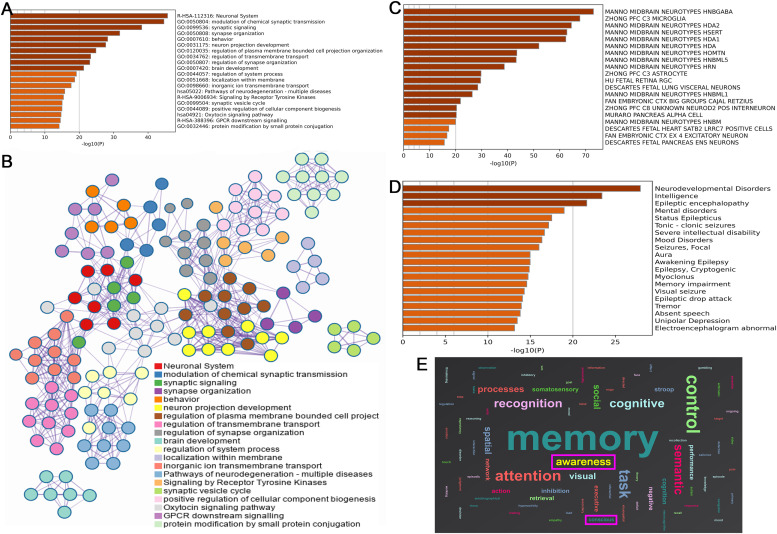
Findings of genes and functional decoding in brain regions with weakened spontaneous brain activities (A. Pathways enrichment analysis; B. Network diagram genes - enrichment pathways; C. Differential genes were co-expressed with the neuronal system; D. Disease enrichment analysis of genes; E. Function decoding results).

A total of 2,080 differentially expressed genes were identified. Enrichment analysis showed that the function of related differential genes was closely related to the synaptic function between neurons, including: neuronal system, modulation of chemical synaptic transmission, synaptic signaling, synapse organization, neuron projection development, and regulation of synapse organization (Fig. 5**. A**). The important results of enrichment analysis suggested that oxytocin signaling pathway was closely related to DOC (Fig. 5**. B**).

Fig. 5. **C** suggested that the related differential genes were co-expressed with the midbrain neuronal system, including: MIDBRAIN NEUROTYPES HNBGABA, HDA2, and HDA1. These diseases related to these genes were electroencephalogram abnormal, status epilepticus, severe intellectual disability, mental disorders, and memory impairment (Fig. 5**. D**). Similar to the results of gene-related disease enrichment, functional decoding showed that the brain regions with weakened spontaneous brain activities mainly involve cognition, attention, memory, awareness and conscious (Fig. 5**. E**).

### Differences of brain activities between wake and DOC groups

**Fig. 6:**
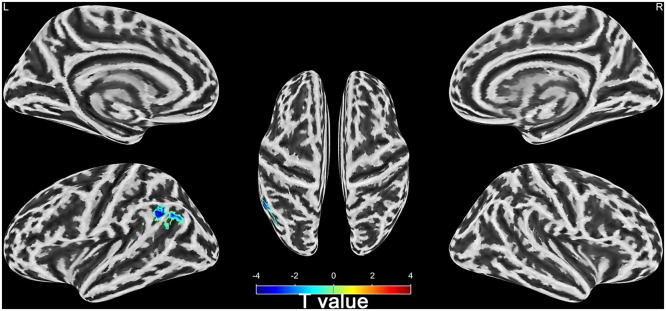
Differences of brain activities between wake and DOC groups (L, left; R, right; Cool tone indicate reduced spontaneous brain activity).

Seven of the 28 sTBI patients with DOC died after 6 months. The remaining 21 patients were divided into wake (n = 13) and DOC groups (n = 8) based on 6-month prognosis. Compared with wake group, the mfALFF of left Angular, Supramarginal Gyrus, and Inferior Parietal Lobule in DOC group was higher (AlphaSim_*P* < 0.01, Cluster > 19, Table 3 and **Supplementary material 3**). Notably, left Angular, Supramarginal Gyrus, and Inferior Parietal Lobule belonged to the left temporoparietal junction region of the DMN (Fig. 6).

**Table 3:** Brain regions with reduced spontaneous brain activity in the wake group compared to the DOC group.

**Voxels**	**Regions**	**Peak MNI x y z**	**T value**	***P*-value**
weakened				
36	Left Angular, Supramarginal Gyrus, Inferior Parietal Lobule	-57, -57, 33	-3.81	AlphaSim < 0.01

MNI, Montreal Neurological Institute

### Quantitative analysis of brain regions with prognostic differences

**Fig. 7:**
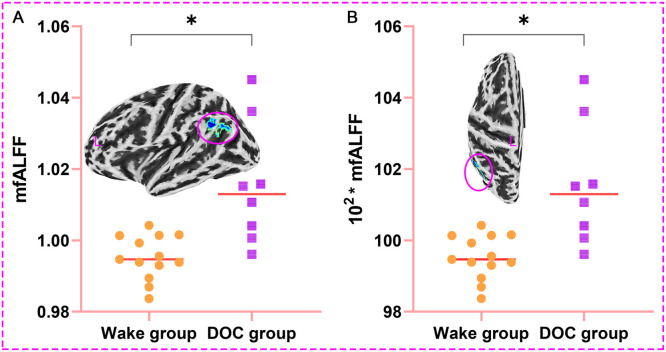
Results of quantitative analysis of brain regions with prognostic differences (*P < 0.05; L, left; A. The mfALFF values of the ROI in the DOC group were higher than in the wake group; B. Result between groups after magnifying the ordinate mfALFF by 100 times; mfALFF, mean fractional amplitude of low frequency fluctuations).

The left Angular, Supramarginal Gyrus, and Inferior Parietal Lobule were used as ROI, and the corresponding mfALFF were extracted for quantitative analysis. We found that mfALFF of ROI in DOC group (1.015±0.017) was statistically higher than that in wake group (0.995±0.006) (T = 3.206, *P* < 0.05, Fig. 7**. A**). Fig. 7**. B** was also the comparison result between groups after magnifying the ordinate mfALFF by 100 times (101.546±1.712 *vs*. 99.529±0.618) to increase the sensitivity of data changes and prepare for the establishment of prediction model (T = 3.206, *P* < 0.05).

### Establishment of Nomogram prediction model based on ROI

**Fig. 8:**
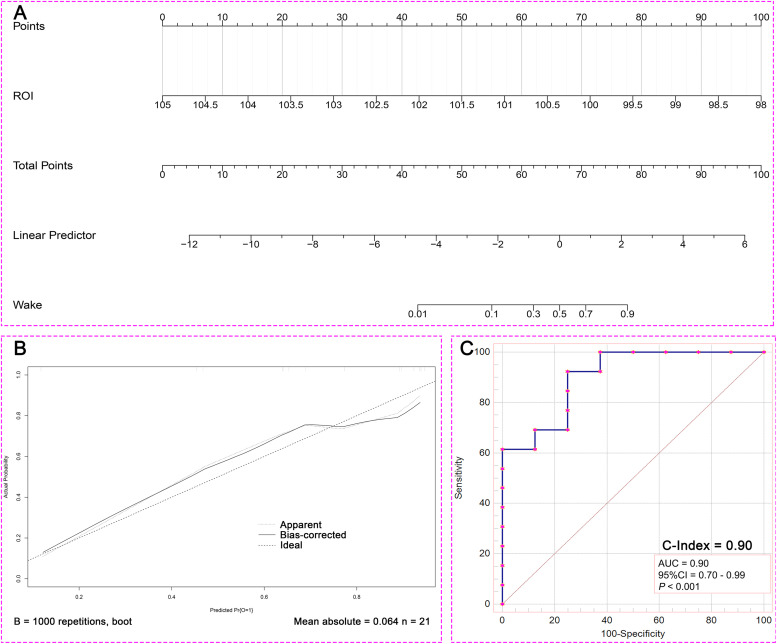
The Nomogram prediction model to assess 6-month prognosis in sTBI patients with DOC (A. Nomogram model; B. Calibration curve; C. Predictive power of Nomogram model; ROI, region of interest; AUC, area under the curve; CI, confidence interval).

Binary Logistic regression analysis was performed using mfALFF values of ROI, and the Nomogram prediction model was established based on R software. Binary Logistic regression showed that the level of spontaneous brain activities in ROI was a prognostic factor (*P* =0.044, Table 4). Fig. 8**. A** was the Nomogram model established based on the mfALFF values of ROI. Fig. 8**. B** was the internal calibration curve and the fluctuation of the calibration curve around the ideal curve indicated that the prediction ability was relatively stable. Specifically, the prediction capacity of the model was shown in Fig. 5. C, and the AUC was 0.90.

**Table 4:** The result of univariate binary Logistic regression analysis.

**Factor**	**β**	**Standard error**	**Wald**	***P*-value**	**OR**	**95%CI**
ROI	-2.771	1.376	4.057	0.044	0.063	0.004 - 0.928

ROI = Left angular gyrus, supramarginal gyrus, and inferior parietal lobule;

OR, odds ratio; CI, confident interval

### Robustness verification of ROI prediction ability

**Fig. 9:**
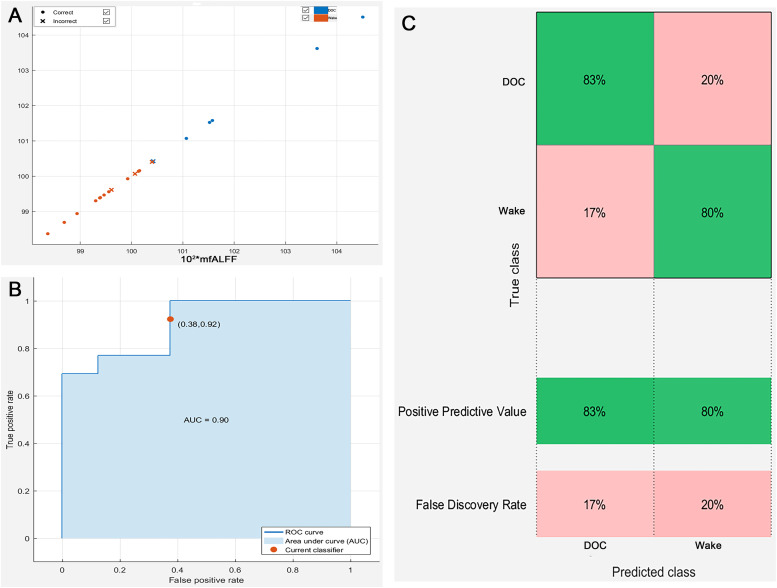
LVSM-based ROI prediction robustness analysis results (A. Scatter plot illustrating the distribution of predictive accuracy data; B. Assessment of AUC for LVSM predictive capability; C.Positive predictive value and false discovery rate of LVMS for predicting patients' six-month prognosis; DOC, disorders of consciousness; mfALFF, mean fractional amplitude of low frequency fluctuations; AUC, area under the curve; ROC, receiver operating characteristic curve).

In order to verify the robustness of ROI prediction ability, the study further used LSVM to identify the prognosis of 21 patients with DOC based on the mfALFF value of ROI. The scatter plot was the data distribution characteristic of predicting the accuracy of consciousness (Fig. 9**. A**). Fig. 9**. B** was the consciousness prediction ability of the LSVM, and the AUC was 0.90. The positive predictive value of LAVM predicting wake at 6 months was 80% and the positive predictive value for DOC was 83% (Fig. 6. C). Based on the brain activities of ROI, both the Nomogram prediction model by binary Logistic regression and the machine learning model based on LVSM showed stable and high prognostic discrimination ability.

## Discussion

The mfALFF which could reflect the spontaneous neural activities was thought to be pathologically and physiologically meaningful (Shen et al., 2019). Firstly, this study aims to explore the differences in brain activity (mfALFF) in 28 sTBI patients with DOC and 30 healthy controls. So far, most sTBI studies have focused on changes in neuronal function and circuitry, which were considered as the basis for DOC associated with sTBI (Xiong et al., 2014). The brain activity of sTBI patients were disrupted, which was closely related to DOC (Thau-Zuchman et al., 2019).

In our study examining the mfALFF values across various brain regions, we discovered that compared to the healthy controls the areas with enhanced brain activities in the DOC patients encompassed several key regions. These regions included the cerebellar tonsil, the posterior and anterior lobes of the cerebellum, the brainstem, thalamus, globus pallidus, limbic lobe, insula, and paracentral lobule. Notably, the regions of heightened brain activities in the brainstem, thalamus, limbic system, and certain cortical areas overlapped significantly with the the ARAS. These areas played a crucial role in the manifestation of consciousness, potentially indicating disturbances within the complex network of neural pathways that regulated arousal (Chen & Chen, 2022; Zhong et al., 2019). The actions of the ARAS primarily positioned in the brainstem, thalamus, limbic system, were paramount in sustaining consciousness, which was also a crucial factor in regulating wakefulness and seamless transition between sleep and arousal (Chen & Chen, 2022; von Gall, 2022). Meanwhile, previous study identified a remarkable escalation in the spontaneous hemodynamic response within the thalamus was discerned in individuals afflicted with unresponsive wakefulness syndrome (UWS), contrasting markedly with the baseline readings observed in a cohort of healthy controls (Wu et al., 2019). This discovery not only underscored the thalamus's critical involvement in the modulation of consciousness, but also served as a crucial biomarker in distinguishing the physiological divergences between UWS patients and those in a state of robust wakefulness. Furthermore, our findings indicate that spontaneous brain activity in the subcortical ARAS is significantly enhanced in sTBI patients, suggesting a disconnection of neural activity from the cortex. Cortical grey matter is sheared away from subcortical white matter and axonal-synaptic connections are forced to break down as a result of the enormous violence of sTBI (Moen et al., 2014; Pendlebury et al., 2022). This type of injury severely impacts the normal function and information transmission of neurons, leading to disruptions in the flow of informational energy. Meanwhile, the ARAS system in the brainstem, as the origin of arousal, continues to emit impulses, resulting in the accumulation of energy and manifesting as excessive activation of the subcortical ARAS (Kayama et al., 1991; Kovalzon, 2016; Moruzzi & Magoun, 1949).

Interestingly, compared with the health controls, the sTBI patients with DOC had a lower mfALFF activation in several brain areas which were mainly located in the default mode network (DMN). The weakened regions included superior frontal gyrus, medial frontal gyrus, inferior frontal gyrus, Brodmann area 10, Brodmann area 46, inferior parietal lobule, superior parietal lobule, supramarginal gyrus, angular gyrus, superior temporal gyrus, middle temporal gyrus, inferior temporal gyrus, precuneus, and posterior cingulate gyrus. The DMN is a cohesive assembly of cortical regions that is now widely recognized as a pivotal component of the brain's functional architecture, mainly composed of brain regions such as the posterior cingulate cortex, anterior cuneiform cortex, medial prefrontal cortex, inferior parietal lobule, and bilateral temporal cortex, playing a fundamental role in maintaining awareness, shaping cognitive, emotional, and self-referential processes (Guillaumin et al., 2018; C. Liu & Yen, 2019; Rode et al., 2022). Based on the required consciousness functions, high-level brain tasks necessitated the DMN and its flexible coupling activities with other networks to achieve the corresponding functions (Kim et al., 2021). The alterations within the DMN might constitute a pivotal factor contributing to DOC and impaired arousal function among sTBI patients (Zhang, et al., 2022). Specifically, the posterior cingulate cortex, angular, supramarginal gyrus, inferior parietal lobule, and part of the prefrontal cortex in the DMN were found to correlate with levels of consciousness (Wolff et al., 2019; Zhang et al., 2017; Zhang, et al., 2022). The state of consciousness in patients with DOC exhibited a profound correlation with the distinct patterns observed within the DMN (Panda et al., 2022). This correlation was strikingly consistent with the findings of our research, which underscores the DMN's pivotal role in the state of consciousness. Previous findings demonstrated a compelling link between the loss of consciousness and the dysfunctional interplay of two pivotal neural circuits. Specifically, the posterior cortical regions, crucial for information transmission, exhibited a failure to relay vital information, coupled with a diminished dissemination of data from subcortical, temporal, parietal, and frontal brain areas (Panda et al., 2023). This observation aligned with our research findings pertaining to the DMN, further reinforcing the coherence and significance of our findings.

Based on the mesocircuit model, the results of the present study reveals a dissociation of spontaneous brain activity in the cortex (represented by the DMN) and in the sub-cortex represented by the ARAS. Specifically, DMN brain activity was consistently lower in DOC patients, whereas ARAS brain activity was significantly higher. Our results suggested that after sTBI, the node efficiency of subcortical ARAS-derived ROIs enhanced. The compensations played a critical role in enhancing arousal, while the node efficiency of cortical DMN-derived ROIs weakened, which might be closely related to deficits in awareness. These results were consistent with the conclusions of the aforementioned studies. It is conceivable that this phenomenon may be attributed to a disruption of cortical and subcortical connections, arousal signal interruptions from the bottom up, resulting in a reverse dissociation of brain activity (Edlow et al., 2021). This precise anatomical segmentation coupled with the segregation of spontaneous brain activity reveals a common mechanism by which sTBI leads to DOC. Remodelling the cortical and subcortical projections could be a promising avenue for improving the level of consciousness. In addition, in the backdrop of the weakened DMN function, we aspired to delve into novel activation strategies in forthcoming research, aiming to establish these approaches as pioneering directions in the future treatment of sTBI. Our research outcomes verify a spectrum of hypotheses and theoretical perspectives that address the emergence of consciousness. Notably, a predominant consensus among these scholarly constructs underscores the indispensable nature of thalamo-cortical connectivity as a cornerstone for the emergence of consciousness (Blumenfeld, 2023; Panda et al., 2022). The mesocircuit hypothesis advanced postulates that the activation of subcortical regions significantly contributes to enhancing cortical function. Previous research has revealed that the metabolic balance of subcortical mesocircuit areas served as a valuable indicator for diagnostic purposes (Annen et al., 2023). Previous studies identified synaptic plasticity within this excitatory corticothalamic circuitry contributed to the facilitation of physiological brain rhythms, thereby playing a pivotal role in the selection process for patients with DOC (Tewarie et al., 2024). The fMRI might offer preliminary and vital evidence supporting the potential existence of disease-specific neural networks (Bharath et al., 2019). Related research testified low-level states of consciousness were marked by disruptions in brain activity that underpinned arousal and awareness, and low-level states of consciousness were correlated with diminished network interactions (Lopez-Gonzalez et al., 2021). This alignment between our findings and the established theories serves to further validate the significance of the dynamic interplay between the thalamus and the cortex in the intricate mechanism of consciousness (Panda et al., 2022).

To further explore this issue, we analyzed the gene matrix from the Allen Human Brain Atlas to identify expressed genes, aiming to clarify the potential biomolecular mechanisms of DOC through gene enrichment results. The study identified a total of 2,080 highly expressed differential genes. Enrichment analysis revealed that the functions of these differential genes were closely related to synaptic function between neurons, including: neuronal system, modulation of chemical synaptic transmission, synaptic signaling, synapse organization, neuron projection development, and regulation of synapse organization (Wertheim et al., 2022). The enrichment analysis findings also suggested that the oxytocin signaling pathway was closely related to synaptic function. The previous study indicated the oxytocin was associated with the depressive disorder, social anxiety disorder and DOC (Fischer & Zilcha-Mano, 2022). In the peripheral system, oxytocin's primary effects involve promoting uterine contractions during childbirth and triggering milk ejection during lactation through its binding to G protein-coupled oxytocin receptors. Within the central nervous system, these same receptors are present in crucial areas such as the cerebral cortex, nucleus accumbens, amygdala, and hippocampus (Jobst et al., 2018; Zilcha-Mano et al., 2021). These cerebral regions exhibit a profound association with the DMN and are intricately linked to the functionality and manifestation of DOC. Oxytocin has garnered significant attention as a biological biomarker, offering predictive insights into psychological disorders, and as a promising therapeutic target for managing various functional psychological disorders (Guastella et al., 2009; Jobst et al., 2018; Zilcha-Mano et al., 2021). The investigation into the relationship between oxytocin and DOC has the potential to emerge as the next frontier in sTBI consciousness disorder research. Simultaneously, oxytocin holds promise as a potent therapeutic agent and target for augmenting DMN brain activities.

Meanwhile, the related differential genes were mainly co-expressed in the midbrain neuron system, including: midbrain neurotypes hindbrain GABA, HDA2, and HDA1. The related differential genes were involved in electroencephalogram abnormal, status epilepticus, severe intellectual disability, mental disorders, and memory impairment. In addition, similar to the enrichment results of genes related diseases, the functions of the brain regions with reduced spontaneous brain activity mainly involved cognition, attention, arousal, and consciousness (Lai et al., 2022; Li et al., 2022). The overexpression of GABAergic neurons, which may be involved in the dysregulation of neurotransmitter homeostasis, reveals the biochemical mechanism of DOC formation (Wu et al., 2020; Yi et al., 2024). GABA neurons are widely distributed throughout the central nervous system (cerebral cortex, hippocampus, thalamus, basal ganglia, brainstem, and cerebellum) and play a crucial role in regulating neuronal excitability (Joshi et al., 2011). Specifically, GABAergic signaling is closely related to consciousness, as it regulates arousal, sleep, cortical synchronization, and conscious perception (Schwartz & Kilduff, 2015; Zhao et al., 2021).

To further understand the DOC patients, we divided them into the wake group and the DOC group according to their prognosis after six months. We discovered differences in spontaneous brain activity among patients with DOC who had varying prognoses. Compared to the DOC group, the wake group had lower spontaneous brain activity in brain areas such as the left angular gyrus, supramarginal gyrus, and inferior parietal lobule. Notably, this differential brain regions belong to the left temporoparietal junction area of the DMN. Excessive local brain activity in DOC group, which implies disruption of long-range connectivity, characterises local energy accumulation (Di Perri et al., 2013; Zhang et al., 2022). This phenomenon is analogous to the generation of new ripples in a confined pool of water, where the energy fails to spread widely but gathers locally, which is indicative of a poor prognosis (Zhang et al., 2022).

The left angular gyrus, supramarginal gyrus, and inferior parietal lobule were used as ROI, and corresponding mfALFF were extracted for quantitative analysis. We found that the mfALFF in the left ROI in the DOC group was significantly higher than the mfALFF in the wake group. The ventral parietal cortex could be divided into its anterior and posterior part, which respectively consist of the supramarginal gyrus and the angular gyrus. The supramarginal gyrus plays a pivotal role in cognitive functions such as reading comprehension, meaning interpretation, and phonological processing. Furthermore, it holds a close association with cognitive impairment and DOC (Kowoll et al., 2016; Rubinstein et al., 2021). The fMRI-backed hypothesis suggested that the supramarginal gyrus primarily handled external and perceptual information, whereas the angular gyrus predominantly processed internal and conceptual information (Daselaar et al., 2013). The left angular gyrus area occupies an important position in the cerebral functional architecture, playing a crucial role in human visual language processing, perceptual information integration, language and thinking processes, and other aspects (Alhajri et al., 2022; Hu et al., 2010). Some studies also found that the angular gyrus regions was closely related to cognitive impairment, disorders of consciousness and regulation of wakefulness state (Zhang et al., 2022; Zhang et al., 2021). sTBI could result in DOC, significantly impeding intracranial communication and transmission (El Youssef et al., 2022; Shen et al., 2023). The crucial elements in reversing DOC lie in the effective activation of the cerebral cortex and the precise regulation of the brain's intricate functional network, not an abnormal aggregation of energy (Shen et al., 2023; Tasserie et al., 2022).

Then, the findings of the binary Logistic regression analysis indicated that the spontaneous brain activity level within the ROI served as a significant prognostic factor. Concurrently, we developed a prediction model for mfALFF values, tailored specifically to the ROI, and subsequently assessed the predictive efficacy of this model. The validation curve exhibited consistent fluctuations around the ideal curve, signaling a relatively dependable predictive capability. Moreover, the predictive power of the model was evidenced by an AUC score of 0.90, which was nearly excellent. This clearly validated the proficiency of our excellent prediction model in accurately forecasting the prognosis of DOC patients. It further offered the clinicians an efficient and precise tool to assess the outlook for DOC patients.

Meanwhile, in the cohort suffering from sTBI with DOC, the LSVM have demonstrated an impressive positive predictive value of 80% in forecasting awakening within six months, as well as a positive predictive value of 83% in forecasting the presence of DOC. These results indicated that both the Nomo prediction model established by binary Logistic regression analysis and the machine learning model established by LSVM exhibited stable and high prognostic discrimination abilities. The excellent performance of these models further validated that these ROI served as the significant biological markers and potential therapeutic targets.

This investigation encountered several noteworthy limitations. Firstly, it was conducted as a study with a relatively constrained sample size. To obtain a more comprehensive understanding, future studies should incorporate a more extensive sample size and longitudinally track the changes across a broader range of disease duration. Secondly, the diverse medications administered to patients with DOC might potentially influence the brain functional activation. One of our forthcoming research priorities involves exploring whether various therapeutic drugs for DOC patients might exert an influence on these cerebral alterations. As we progress in our research, we would also enhance the management of treatment protocols to further mitigate interfering factors. Thirdly, in light of the functional attribute findings, it would be beneficial to corroborate and complement the conclusions by providing information on structural scales and further study is necessary in the future.

## Conclusions

This study could enhance our understanding of DOC in patients with sTBI by revealing the dissociation between cortical and subcortical spontaneous brain activities. The findings demonstrated significant alterations in spontaneous brain activity within the mesocircuit, specifically involving the ARAS and DMN. These regions were crucial for consciousness regulation and were disrupted in DOC patients. Additionally, gene enrichment analyses highlighted the involvement of synaptic function, the oxytocin signaling pathway, and GABAergic processes in the formation of DOC. The predictive models based on mfALFF data showed impressive accuracy in forecasting recovery of consciousness, offering a promising tool for clinical prognosis. Overall, this study supported the clinical utility of mfALFF as a robust and non-invasive measure for assessing brain functions and predicting recovery outcomes in sTBI patients with DOC.

## Informed consent

Informed consents were obtained from patients’ family members and healthy participants.

## Consent to publish

All authors have reviewed this manuscript and consented to its publication.

## Ethical compliance statement

This study was approved by the Medical Ethics Committee (2020KY-179). All procedures performed were in accordance with the ethical standards of the institutional committee.

## Role of funding source

Project of Chongqing Key Laboratory of Emergency Medicine, Grant/Award Number: 2024RCCX01; 10.13039/501100012226Fundamental Research Funds for the Central Universities, Grant/Award Number: 2023CDJYGRH-YB09; 10.13039/501100012669Natural Science Foundation Project of Chongqing, Grant/Award Number: CSTB2023NSCQ-BHX0074; Key Project of Science and Technology Research Program of 10.13039/501100007957Chongqing Municipal Education Commission: KJZD-K202400106; and Hospital Level Support Projects, Grant/Award Number: Fcjs202050.

## Supplementary material

Supplementary material associated with this article can be found, in the online version.

## CRediT authorship contribution statement

**Chang Li:** Conceptualization, Visualization, Writing – original draft, Writing – review & editing. **Peng Chen:** Conceptualization, Visualization, Writing – original draft, Writing – review & editing. **Yongbing Deng:** Writing – original draft, Writing – review & editing. **Lei Xia:** Writing – original draft, Writing – review & editing. **Xiaodong Wang:** Writing – original draft, Writing – review & editing. **Min Wei:** Writing – original draft, Writing – review & editing. **Xingdong Wang:** Writing – original draft, Writing – review & editing. **Lun Dong:** Conceptualization, Visualization, Data curation, Writing – review & editing. **Jun Zhang:** Conceptualization, Visualization, Data curation, Formal analysis, Writing – original draft, Writing – review & editing.

## Declaration of competing interest

The authors report no conflicts of interest in this work.

## Appendix Supplementary materials

Image, application 1

Image, application 2

Image, application 3

Image, application 4
