# Supplementary material for: Abnormalities of cortical and subcortical spontaneous brain activity unveil mechanisms of disorders of consciousness and prognosis in patients with severe traumatic brain injury
Source: Int J Clin Health Psychol. 2024 Nov 28;24(4):100528. doi: 10.1016/j.ijchp.2024.100528 (PMC11629552; doi:10.1016/j.ijchp.2024.100528)
Supplement: Supplementary file 1 [file mmc1.doc]

**Supplementary material 1**. Summary of brain region results for increased spontaneous brain activity in DOC patients relative to healthy controls.

This report is based on CUI Xu's xjview. (http://www.alivelearn.net/xjview/)

Revised by YAN Chao-Gan and ZHU Wei-Xuan 20091108: suitable for different Cluster Connectivity Criterion: surface connected, edge connected, corner connected.

Number of clusters found: 3

----------------------

Cluster 1

Number of voxels: 382

Peak MNI coordinate: 6 -39 -33

Peak MNI coordinate region: // Right Brainstem // Pons // undefined // undefined // undefined // undefined

Peak intensity: 9.2452

# voxels structure

382 --TOTAL # VOXELS--

193 Cerebellum Posterior Lobe

157 Left Cerebellum

141 Right Cerebellum

105 Cerebellar Tonsil

104 Cerebellum Anterior Lobe

72 Pons

63 Cerebelum_8_L (aal)

48 Cerebelum_8_R (aal)

48 Pyramis

39 Left Brainstem

39 Right Brainstem

30 Cerebelum_9_L (aal)

22 Inferior Semi-Lunar Lobule

20 Dentate

18 Culmen

14 Cerebelum_9_R (aal)

11 Uvula

11 Cerebelum_7b_L (aal)

10 Nodule

8 Declive

8 Cerebelum_Crus1_L (aal)

8 Cerebelum_6_R (aal)

6 Medulla

5 Vermis_10 (aal)

5 Vermis_8 (aal)

5 Cerebelum_4_5_L (aal)

4 Cerebelum_3_R (aal)

3 Cerebelum_Crus2_L (aal)

1 Cerebelum_Crus2_R (aal)

1 Sub-lobar

1 Fourth Ventricle

1 Cerebro-Spinal Fluid

1 Cerebelum_Crus1_R (aal)

1 Cerebelum_6_L (aal)

----------------------

Cluster 2

Number of voxels: 226

Peak MNI coordinate: -6 -21 -12

Peak MNI coordinate region: // Left Brainstem // Midbrain // undefined // undefined // undefined // undefined

Peak intensity: 7.4753

# voxels structure

226 --TOTAL # VOXELS--

157 Left Cerebrum

128 Sub-lobar

121 White Matter

82 Extra-Nuclear

47 Left Brainstem

45 Midbrain

39 Gray Matter

25 Frontal Lobe

23 Thalamus_L (aal)

22 Cerebellum Anterior Lobe

22 Left Cerebellum

20 Culmen

20 Sub-Gyral

17 Lentiform Nucleus

15 Thalamus

14 Cerebelum_3_L (aal)

13 Cerebelum_4_5_L (aal)

12 Insula

10 Putamen

7 Putamen_L (aal)

4 Ventral Posterior Lateral Nucleus

4 Lateral Globus Pallidus

4 Ventral Lateral Nucleus

3 Precentral Gyrus

3 Precentral_L (aal)

3 Medial Globus Pallidus

3 Postcentral Gyrus

2 Pallidum_L (aal)

2 Pons

2 Insula_L (aal)

2 Claustrum

2 Limbic Lobe

2 Pulvinar

2 Cerebellar Lingual

2 brodmann area 13

2 Cingulate Gyrus

1 Optic Tract

1 Inferior Parietal Lobule

1 Ventral Anterior Nucleus

1 Parietal Lobe

1 Medial Geniculum Body

1 Substania Nigra

1 Subthalamic Nucleus

1 Temporal Lobe

----------------------

Cluster 3

Number of voxels: 128

Peak MNI coordinate: 30 -27 21

Peak MNI coordinate region: // Right Cerebrum // Sub-lobar // Extra-Nuclear // White Matter // undefined // undefined

Peak intensity: 5.7809

# voxels structure

128 --TOTAL # VOXELS--

128 Right Cerebrum

118 White Matter

53 Sub-lobar

42 Frontal Lobe

37 Extra-Nuclear

32 Limbic Lobe

31 Cingulate Gyrus

26 Sub-Gyral

14 Insula

13 Medial Frontal Gyrus

11 Cingulum_Mid_R (aal)

10 Gray Matter

6 Insula_R (aal)

4 brodmann area 13

4 Supp_Motor_Area_R (aal)

2 Inferior Parietal Lobule

2 Paracentral Lobule

2 brodmann area 31

2 Thalamus_R (aal)

1 Rolandic_Oper_R (aal)

1 Parietal Lobe

1 Postcentral Gyrus

1 brodmann area 24

1 Claustrum

1 brodmann area 6

1 Thalamus

>>
