# Supplementary material for: Abnormalities of cortical and subcortical spontaneous brain activity unveil mechanisms of disorders of consciousness and prognosis in patients with severe traumatic brain injury
Source: Int J Clin Health Psychol. 2024 Nov 28;24(4):100528. doi: 10.1016/j.ijchp.2024.100528 (PMC11629552; doi:10.1016/j.ijchp.2024.100528)
Supplement: Supplementary file 2 [file mmc2.doc]

**Supplementary material 2**. Summary of results for brain regions with reduced spontaneous brain activity in DOC patients compared with healthy controls.

This report is based on CUI Xu's xjview. (http://www.alivelearn.net/xjview/)

Revised by YAN Chao-Gan and ZHU Wei-Xuan 20091108: suitable for different Cluster Connectivity Criterion: surface connected, edge connected, corner connected.

Number of clusters found: 6

----------------------

Cluster 1

Number of voxels: 407

Peak MNI coordinate: 3 57 -15

Peak MNI coordinate region: // Right Cerebrum // Frontal Lobe // Medial Frontal Gyrus // Gray Matter // brodmann area 11 // Rectus_R (aal)

Peak intensity: -8.1651

# voxels structure

407 --TOTAL # VOXELS--

365 Frontal Lobe

313 Right Cerebrum

192 Gray Matter

157 White Matter

128 Medial Frontal Gyrus

103 Superior Frontal Gyrus

97 Middle Frontal Gyrus

94 brodmann area 11

86 brodmann area 10

72 Frontal_Mid_R (aal)

56 Frontal_Sup_R (aal)

52 Left Cerebrum

49 Rectus_L (aal)

48 Rectus_R (aal)

46 Frontal_Med_Orb_R (aal)

43 Frontal_Mid_Orb_R (aal)

37 Inter-Hemispheric

37 Frontal_Med_Orb_L (aal)

36 Frontal_Sup_Orb_R (aal)

16 Frontal_Sup_Medial_R (aal)

15 Inferior Frontal Gyrus

13 Orbital Gyrus

7 brodmann area 46

6 Rectal Gyrus

3 Sub-Gyral

----------------------

Cluster 2

Number of voxels: 906

Peak MNI coordinate: 60 -42 36

Peak MNI coordinate region: // Right Cerebrum // Parietal Lobe // Supramarginal Gyrus // undefined // undefined // SupraMarginal_R (aal)

Peak intensity: -7.7085

# voxels structure

906 --TOTAL # VOXELS--

845 Right Cerebrum

596 Parietal Lobe

407 Gray Matter

387 Inferior Parietal Lobule

371 White Matter

285 SupraMarginal_R (aal)

247 Temporal Lobe

234 brodmann area 40

174 Angular_R (aal)

155 Parietal_Inf_R (aal)

134 Temporal_Mid_R (aal)

106 Middle Temporal Gyrus

103 Superior Temporal Gyrus

92 Supramarginal Gyrus

70 brodmann area 39

66 Postcentral Gyrus

56 Temporal_Sup_R (aal)

48 Angular Gyrus

48 Occipital_Mid_R (aal)

24 brodmann area 7

24 brodmann area 22

22 Parietal_Sup_R (aal)

20 Superior Parietal Lobule

17 Precuneus

16 brodmann area 2

15 brodmann area 21

8 Temporal_Inf_R (aal)

6 brodmann area 1

5 brodmann area 3

4 brodmann area 19

4 brodmann area 37

3 Inferior Temporal Gyrus

2 Postcentral_R (aal)

1 Precentral Gyrus

1 Occipital_Sup_R (aal)

1 Occipital Lobe

1 brodmann area 13

1 brodmann area 4

1 Sub-Gyral

1 brodmann area 43

1 Superior Occipital Gyrus

1 Frontal Lobe

----------------------

Cluster 3

Number of voxels: 155

Peak MNI coordinate: -45 39 21

Peak MNI coordinate region: // Left Cerebrum // Frontal Lobe // Middle Frontal Gyrus // Gray Matter // brodmann area 46 // Frontal_Mid_L (aal)

Peak intensity: -5.8242

# voxels structure

155 --TOTAL # VOXELS--

155 Frontal Lobe

155 Left Cerebrum

103 Frontal_Inf_Tri_L (aal)

96 White Matter

84 Middle Frontal Gyrus

66 Inferior Frontal Gyrus

54 Gray Matter

47 Frontal_Mid_L (aal)

33 brodmann area 46

12 brodmann area 10

5 Sub-Gyral

4 brodmann area 45

3 Frontal_Inf_Orb_L (aal)

2 brodmann area 47

----------------------

Cluster 4

Number of voxels: 858

Peak MNI coordinate: 0 -60 33

Peak MNI coordinate region: // Left Cerebrum // Parietal Lobe // Precuneus // undefined // undefined // Precuneus_L (aal)

Peak intensity: -8.2438

# voxels structure

858 --TOTAL # VOXELS--

507 Precuneus

478 Left Cerebrum

454 Parietal Lobe

365 Gray Matter

323 Right Cerebrum

307 Precuneus_L (aal)

301 White Matter

242 Precuneus_R (aal)

240 Limbic Lobe

192 brodmann area 7

129 Posterior Cingulate

123 brodmann area 31

116 Cingulate Gyrus

95 Occipital Lobe

82 Cuneus_L (aal)

57 Inter-Hemispheric

52 Cingulum_Mid_L (aal)

48 Calcarine_L (aal)

38 Cingulum_Post_L (aal)

28 Cuneus_R (aal)

25 Cuneus

22 brodmann area 23

17 brodmann area 30

17 Cingulum_Post_R (aal)

16 Calcarine_R (aal)

16 Sub-Gyral

15 Cingulum_Mid_R (aal)

9 brodmann area 29

6 Temporal Lobe

5 Extra-Nuclear

5 Sub-lobar

4 Frontal Lobe

4 Paracentral Lobule

2 brodmann area 5

1 Superior Parietal Lobule

1 Corpus Callosum

----------------------

Cluster 5

Number of voxels: 313

Peak MNI coordinate: -63 -36 33

Peak MNI coordinate region: // Left Cerebrum // Parietal Lobe // Inferior Parietal Lobule // Gray Matter // brodmann area 40 // SupraMarginal_L (aal)

Peak intensity: -9.4539

# voxels structure

313 --TOTAL # VOXELS--

302 Left Cerebrum

229 Parietal Lobe

173 Gray Matter

157 Inferior Parietal Lobule

131 brodmann area 40

115 SupraMarginal_L (aal)

102 White Matter

80 Parietal_Inf_L (aal)

72 Temporal Lobe

55 Supramarginal Gyrus

52 Superior Temporal Gyrus

37 Temporal_Mid_L (aal)

30 Postcentral Gyrus

18 brodmann area 39

17 Angular_L (aal)

12 Temporal_Sup_L (aal)

10 brodmann area 22

10 Postcentral_L (aal)

8 Middle Temporal Gyrus

8 brodmann area 2

3 brodmann area 42

2 brodmann area 1

1 Occipital Lobe

1 brodmann area 19

----------------------

Cluster 6

Number of voxels: 101

Peak MNI coordinate: -39 -75 24

Peak MNI coordinate region: // Left Cerebrum // Temporal Lobe // Middle Temporal Gyrus // Gray Matter // brodmann area 39 // Occipital_Mid_L (aal)

Peak intensity: -6.5802

# voxels structure

101 --TOTAL # VOXELS--

101 Left Cerebrum

77 Parietal Lobe

54 White Matter

47 Gray Matter

39 Angular_L (aal)

29 Occipital_Mid_L (aal)

24 Inferior Parietal Lobule

23 brodmann area 39

23 Superior Parietal Lobule

22 Temporal Lobe

20 Middle Temporal Gyrus

20 Angular Gyrus

17 brodmann area 7

16 Parietal_Inf_L (aal)

13 Parietal_Sup_L (aal)

11 Precuneus

6 brodmann area 40

2 Temporal_Mid_L (aal)

2 Occipital Lobe

2 Superior Occipital Gyrus

1 Sub-Gyral

1 brodmann area 19

>>
