# Supplementary material for: Abnormalities of cortical and subcortical spontaneous brain activity unveil mechanisms of disorders of consciousness and prognosis in patients with severe traumatic brain injury
Source: Int J Clin Health Psychol. 2024 Nov 28;24(4):100528. doi: 10.1016/j.ijchp.2024.100528 (PMC11629552; doi:10.1016/j.ijchp.2024.100528)
Supplement: Supplementary file 3 [file mmc3.doc]

**Supplementary material 3**. Outcome set of brain regions with enhanced spontaneous brain activity in the DOC group compared to the wake group after 6-month.

This report is based on CUI Xu's xjview. (http://www.alivelearn.net/xjview/)

Revised by YAN Chao-Gan and ZHU Wei-Xuan 20091108: suitable for different Cluster Connectivity Criterion: surface connected, edge connected, corner connected.

Number of clusters found: 1

----------------------

Cluster 1

Number of voxels: 36

Peak MNI coordinate: -57 -57 33

Peak MNI coordinate region: // Left Cerebrum // Parietal Lobe // Supramarginal Gyrus // Gray Matter // brodmann area 40 // Angular_L (aal)

Peak intensity: -3.8058

# voxels structure

36 --TOTAL # VOXELS--

36 Left Cerebrum

36 Parietal Lobe

26 Angular_L (aal)

24 White Matter

17 Supramarginal Gyrus

12 Gray Matter

11 brodmann area 40

10 Angular Gyrus

9 Inferior Parietal Lobule

7 Parietal_Inf_L (aal)

1 brodmann area 39

>>
