# Supplementary material for: Abnormalities of cortical and subcortical spontaneous brain activity unveil mechanisms of disorders of consciousness and prognosis in patients with severe traumatic brain injury
Source: Int J Clin Health Psychol. 2024 Nov 28;24(4):100528. doi: 10.1016/j.ijchp.2024.100528 (PMC11629552; doi:10.1016/j.ijchp.2024.100528)
Supplement: Supplementary file 4 [file mmc4.doc]

**Supplementary table. Behavioural characteristics of included sTBI patients.**

| **Number** | **Age** | **Gender** | **Right-handed** | **Education** | **Trauma etiology** | **Past medical** | **Onset to data collection time** | **Admission GCS score** | **CRS-R score**  **at fMRI** | **GOS-E score**  **at 6-month** |
| --- | --- | --- | --- | --- | --- | --- | --- | --- | --- | --- |
| 1 | 73 | Male | Yes | 6 | Traffic accident | - | 7 | 5 | 3 | 1 |
| 2 | 68 | Female | Yes | 3 | Traffic accident | Hypertension | 28 | 8 | 4 | 1 |
| 3 | 63 | Male | Yes | 5 | Traffic accident | - | 15 | 2T | 0 | 1 |
| 4 | 74 | Male | Yes | 7 | Traffic accident | - | 22 | 5 | 3 | 1 |
| 5 | 49 | Female | Yes | 10 | Traffic accident | - | 11 | 5 | 3 | 1 |
| 6 | 59 | Female | Yes | 4 | Traffic accident | Hypertension | 25 | 8 | 5 | 1 |
| 7 | 64 | Male | Yes | 10 | Traffic accident | Diabetes | 9 | 8 | 6 | 1 |
| 8 | 68 | Male | Yes | 5 | Traffic accident | Hypertension | 27 | 8 | 9 | 7 |
| 9 | 63 | Female | Yes | 8 | Traffic accident | Hypertension and diabetes | 13 | 2T | 4 | 7 |
| 10 | 64 | Male | Yes | 4 | Traffic accident | - | 18 | 5 | 6 | 5 |
| 11 | 79 | Female | Yes | 6 | Traffic accident | - | 8 | 8 | 6 | 4 |
| 12 | 73 | Male | Yes | 2 | Traffic accident | - | 24 | 8 | 5 | 3 |
| 13 | 62 | Male | Yes | 12 | Falls from height | - | 16 | 8 | 8 | 6 |
| 14 | 44 | Male | Yes | 14 | Traffic accident | - | 10 | 3 | 3 | 3 |
| 15 | 64 | Male | Yes | 8 | Traffic accident | - | 24 | 5 | 6 | 5 |
| 16 | 72 | Female | Yes | 6 | Traffic accident | Hypertension | 14 | 6 | 5 | 3 |
| 17 | 80 | Male | Yes | 3 | Traffic accident | Hypertension and diabetes | 19 | 5 | 3 | 4 |
| 18 | 49 | Male | Yes | 8 | Traffic accident | Hypertension | 12 | 2T | 3 | 3 |
| 19 | 71 | Female | Yes | 5 | Traffic accident | - | 23 | 8 | 8 | 6 |
| 20 | 33 | Female | Yes | 14 | Traffic accident | Asthma | 20 | 8 | 6 | 5 |
| 21 | 30 | Male | Yes | 12 | Traffic accident | - | 17 | 6 | 4 | 2 |
| 22 | 44 | Male | Yes | 8 | Traffic accident | - | 21 | 2T | 0 | 2 |
| 23 | 42 | Male | Yes | 6 | Traffic accident | - | 28 | 3 | 2 | 2 |
| 24 | 18 | Male | Yes | 12 | Traffic accident | - | 7 | 2T | 0 | 2 |
| 25 | 52 | Male | Yes | 3 | Traffic accident | - | 24 | 2T | 0 | 2 |
| 26 | 46 | Male | Yes | 11 | Traffic accident | - | 15 | 3 | 2 | 2 |
| 27 | 53 | Male | Yes | 7 | Traffic accident | Diabetes | 26 | 3 | 2 | 2 |
| 28 | 57 | Female | Yes | 8 | Traffic accident | Hypertension and diabetes | 11 | 2T | 0 | 2 |

GCS, Glasgow Coma Scale; CRS-R, Coma Recovery Scale - Revised; fMRI, functional Magnetic Resonance Imaging; GOS-E, Glasgow Outcome Scale - Extended; T, Tracheostomy
